# Management of Ureteric Small Bowel Fistula: A Case Report and Review of the Current Literature

**DOI:** 10.1155/criu/7232300

**Published:** 2025-08-08

**Authors:** Panagiota Fallon, Melissa Matthews, Abhisekh Chatterjee, Dimitrios Sapountzis, Nikolaos Chatzikrachtis, Katie McComb, Samuel Bishara, Ivo Donkov, Konstantinos Charitopoulos, Panagiotis Nikolinakos

**Affiliations:** ^1^Department of Urology, West Middlesex University Hospital, Chelsea and Westminster Hospital NHS Foundation Trust, London, UK; ^2^Department of Medicine, Faculty of Medicine, Imperial College London, London, UK; ^3^Department of Surgery, West Middlesex University Hospital, Chelsea and Westminster NHS Foundation Trust, London, UK

**Keywords:** case report, conservative management, ureteric small bowel fistula, urology

## Abstract

**Background:** Ureteric small bowel fistulas are rare entities, with limited reports in the literature. These pathological connections between the ureter and small bowel can lead to recurrent upper urinary tract infections and pose significant diagnostic and therapeutic challenges. The primary cause of ureteric small bowel fistula formation is iatrogenic intervention, such as percutaneous nephrolithotomy (PCNL) or abdominal surgery involving tissue resection. However, they can also arise spontaneously due to local chronic inflammation and infection. Given their rarity, there is no standardised management pathway, and the treatment approach should be individualised.

**Case Presentation:** We present the case of a 57-year-old man presenting with a history of extensive locally advanced distal sigmoid carcinoma, managed with Hartmann's procedure with end colostomy followed by adjuvant radiotherapy and chemotherapy. He later developed a mid-ureteric small bowel fistula, presenting with urosepsis, a high-output stoma, deteriorating renal function and severe metabolic acidosis. Conservative management with regular ureteric stent exchanges every 3–4 months has successfully preserved renal function and improved his quality of life. Surgical closure was not pursued due to high operative risk, and repeat imaging has shown no evidence of persisting fistula, suggesting possible spontaneous closure.

**Conclusion:** The treatment options for this ureteric small bowel fistula were limited. A lifelong nephrostomy was deemed unsuitable due to incompatibility with the patient's profession, and surgical intervention was associated with significant complexity due to the patient's history of malignancy and radiotherapy-related tissue changes. Therefore, a conservative strategy involving serial ureteric stent exchanges was pursued. Early recognition and individualised treatment of ureteric-enteric fistulas are essential, as timely intervention can significantly enhance prognosis and quality of life.

## 1. Introduction

Ureteric fistulas are rare abnormal connections between the ureter and neighbouring structures, such as the bowel, arteries or skin. These fistulas present significant diagnostic and management challenges due to their nonspecific and variable clinical presentations, which depend on the fistula's size, location and underlying aetiology. Symptoms may mimic more common urological or gastrointestinal conditions, leading to delays in diagnosis [1]. They usually not only are a result of iatrogenic interventions [2–5] but also can form due to malignancy [2], inflammation [6, 7] and infections [8, 9], or a combination of those. Therapeutic decisions are further complicated by the lack of consensus or guidelines on optimal management, particularly in patients with complex surgical or oncological histories. This case report presents a ureteric small bowel fistula that developed following anterior resection for carcinoma of the distal sigmoid colon. Although prior cases of similar fistulas have been documented, management strategies are limited in the literature.

## 2. Case Presentation

A 57-year-old man with a diagnosis of nonmetastatic locally advanced distal sigmoid carcinoma underwent anterior resection with total mesorectal excision (TME) and Hartmann's procedure with end-colostomy fashioning. The location of the tumour and extent of disease warranted a concomitant bladder cuff removal and bilateral ureteric stent placement for tumour-related hydronephrosis. Histopathology showed moderately differentiated adenocarcinoma, pT4a N0, R1 resection, with extramural venous invasion (EMVI+), but no evidence of malignancy at the bladder cuff resection margin. The patient received adjuvant chemotherapy (12 cycles of FOLFOX) starting 1 month postoperatively and subsequent pelvic chemoradiotherapy. This was indicated due to a microscopic positive margin at the left pelvic sidewall and clinical concern for bladder involvement, although the latter was not proven histologically.

For nearly 2 years, the patient remained well, with serial CT scans and an MRI of the pelvis showing no evidence of local complications. During this period, ureteric stents were exchanged every 3 months due to prior ureteric obstruction.

The patient presented with acute worsening of renal function, recurrent urinary tract infections (rUTIs) over a few weeks, and a high-output stoma (> 1.5 L/day), 2 years after surgery. Symptoms also included oliguria (< 1 L per day or < 50 mL per hour), nausea and vomiting.

Initial investigations revealed severe dehydration with profound metabolic acidosis (pH 7.15, base excess (BE): −22), hypokalaemia (K+ 2.5 mmol/L), and acute kidney injury (AKI) (creatinine 352 *μ*mol/L, urea 49 mmol/L, and estimated glomerular filtration rate (eGFR) of 16).

Analysis of colostomy output revealed a urine-like fluid with markedly elevated creatinine (5352 *μ*mol/L), urea (46.9 mmol/L), Na+ (105 mmol/L), and K+ (13 mmol/L), suggesting a urointestinal fistula. He was admitted to the high dependency unit (HDU) for monitored fluid resuscitation and electrolyte correction.

## 3. Management Strategy

The patient received intravenous fluids and bicarbonate supplementation per high-output stoma protocol. Colostomy output initially measured 2 L in 24 h, and with rehydration, it increased up to 6 L in 24 h. In contrast, urinary output remained at approximately 1 L per day, which also supports that there was diversion of urine.

**Figure 1:**
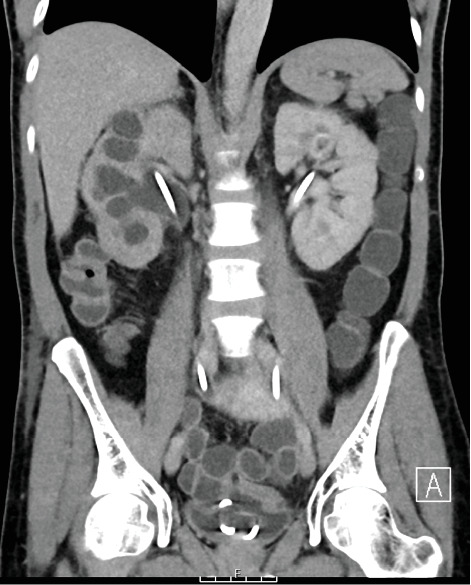
Initial CT urogram (coronal plane) which demonstrates the bilateral stents in situ. A portion of the small bowel where the contrast has extravasated is seen. There is also right pelvicalyceal dilatation.

**Figure 2:**
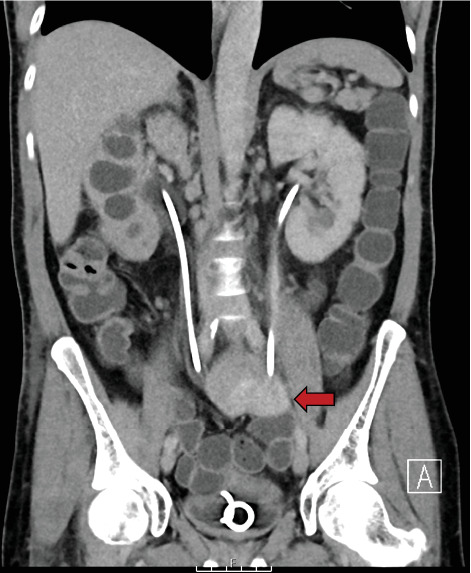
Initial CT urogram (coronal plane). A further portion of the small bowel with contrast extravasation can be seen. The left perinephric stranding is also visible in this image.

**Figure 3:**
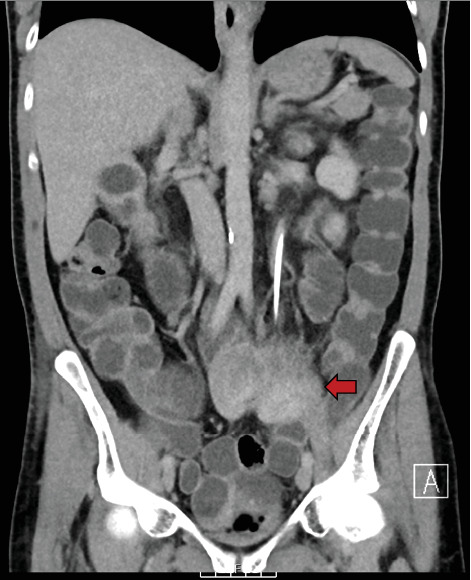
Initial CT urogram (coronal plane). Extravasation of contrast into the small bowel and fluid-filled small and large bowel.

An urgent CT (computed tomography) with urographic phase of contrast was performed (CT urogram), which revealed extravasation of contrast from the left ureter into the small bowel (Figures 1, 2, and 3). The fistula was located at the level of S1, involving the mid-left ureter and a segment of terminal ileum. The tract appeared to be side-to-side in nature.

Initial management included left nephrostomy insertion to divert urine flow away from the fistula. This resulted in a significant reduction in stoma output and improvement in the patient's metabolic status, including resolution of acidosis, normalisation of renal function and clinical stabilisation.

**Figure 4:**
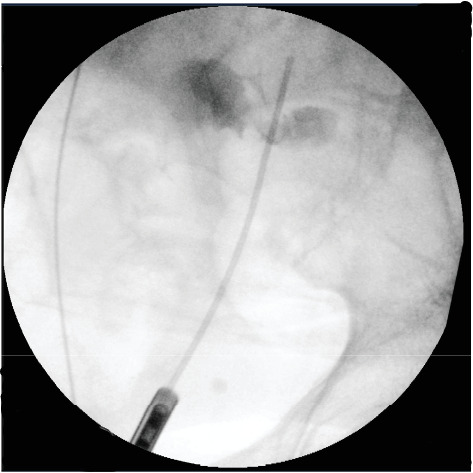
Intraoperative retrograde study on the left during ureteric stent exchange, demonstrating extravasation of contrast from the mid-left ureter (vertebral level S1) to the small bowel.

Subsequently, the patient underwent bilateral JJ stent exchange, as he already had previous ureteric stents in situ. The retrograde study on the left demonstrated a fistula between the left ureter and the small bowel (Figure 4). The nephrostomy was maintained for approximately 2 months, after which an antegrade nephrostogram demonstrated restored ureteric flow without contrast leakage. Ureteroscopy was considered but ultimately not performed, as the diagnosis had been adequately established radiologically, and the clinical course was favourable with diversion and stenting alone.

Given the patient's history of malignancy, history of radiotherapy and the technical complexity associated with surgical intervention, invasive surgical repair was not pursued. Additionally, lifelong nephrostomy was deemed unsuitable due to occupational considerations of the patient. Instead, a conservative management strategy was adopted, involving routine ureteric stent exchanges every 3–4 months, regular reviews in oncology, colorectal surgery and urology clinics. Nearly 3 years since the fistula was identified, the patient remains asymptomatic, with stable renal function, no recurrence of the fistula and no evidence of oncologic relapse. He continues to undergo regular ureteric stent exchanges without complications so far.

## 4. Discussion

This case illustrates the challenges associated with the diagnosis and management of ureteric small bowel fistulas, particularly in patients with a history of pelvic malignancy and prior surgery.

The patient's biochemical findings are of particular interest: Before nephrostomy placement, the colostomy output exhibited biochemical characteristics consistent with urine, suggesting that the fistula had temporarily relieved ureteric obstruction. Interestingly, his eGFR improved with rehydration even before nephrostomy insertion, likely because small bowel efficiently drains urine, similar to its role in urinary diversions. However, the small bowel is not an optimal conduit for all urinary components, particularly chloride, which is reabsorbed, leading to hyperchloremic acidosis, an established complication of ileal conduits. In this case, the severity of acidosis likely reflects prolonged urine exposure over a greater bowel segment than typically observed in ileal conduits (where the length of small bowel used is around 20 cm). A comparable situation was described by Banshodani et al. (2017), where an artificial ureteroenteric fistula was fashioned as a last resort for encapsulating peritoneal sclerosis, where other surgeries were deemed unsuitable due to severe abdominal adhesions [10].

In this current case, the potential cause of the ureteric small bowel fistula formation is speculated to be due to a combination of previous radiotherapy, previous surgical manipulations and chronic ureteric stenting, which may have contributed to local ischemia and tissue fragility and breakdown. Radiation has long been associated with delayed tissue necrosis, ureteric strictures and fistula formation, even years after treatment. It has also been observed that other urological conditions may lead to fistula formation, such as urinary tract stones, especially staghorn calculi that require PCNL [11], and rUTIs. It is important to highlight that conservative management of ureteroenteric fistulas carries significant long-term risks. Even with a ureteric stent in place, rUTIs may persist and can lead to severe complications, including sepsis, which can prove life-threatening. Ureteric stents typically require replacement at regular intervals (usually every 3–6 months), a procedure that often necessitates the use of anaesthesia. Patients that are selected for conservative management are frequently those who are deemed high risk for surgical intervention, which can further complicate ongoing care when regular stent exchanges are needed. It is therefore in the hands of the clinician to carefully assess the benefits and risks of a potential intervention, with an individualised approach to the patient. Possible surgical interventions for such fistulas include fistula tract excision with ureteral reconstruction techniques, such as ureteroureterostomy or ureteroneocystostomy, sometimes combined with bowel segment resection. In complex cases, urinary diversion or tissue flap interposition may be necessary to ensure healing and prevent recurrence [12]. These are complex surgeries that require careful consideration and should be approached with caution, especially in patients with prior malignancy, radiation or other comorbidities.

In the literature review that we conducted, there were limited previous cases identified that describe ureteric small bowel fistulas (Table 1). In Chang et al.'s study, conservative management was pursued, where the patient recovered well and was managed with regular JJ stent exchanges [3]. We also identified four case reports where the patients were managed surgically. Appleyard and Jones described a ureteric small bowel fistula that developed after the repair of a ureteric injury sustained during an abdomino-perineal excision. The fistula was successfully managed with a repeat laparotomy and Boari flap reconstruction of the ureter [4]. Blumgart and Thakur performed a diagnostic laparotomy with ureteric re-implantation and right hemicolectomy to treat a uretero-ileal fistula in a patient with Crohn's disease [6]. Critchley et al. reported an emergency laparotomy for the repair of a ureteric small bowel fistula in a patient with pyelocystitis and rUTIs. The condition was later managed with an elective nephro-ureterectomy [8]. Scott described a ureteric small bowel fistula in a patient presenting with lower urinary tract symptoms (LUTSs), abdominal and flank pain. The fistula was surgically managed with a nephro-ureterectomy due to significant pyonephrosis, along with small bowel resection [9]. The case by Mir et al. [7] highlights how urological complications can arise from nonurological conditions, such as peptic ulcers, due to their anatomical proximity to the urinary tract. In that case, the fistula was managed with a nephrostomy to divert urine, similar to the approach in our patient. In contrast, the report by Liu et al. [5] demonstrates how complications from common urological interventions, specifically misplacement of a JJ stent through a urostomy, can lead to rare but severe outcomes like a uretero-ileal fistula. Tragically, the patient died from septic shock before definitive management could be pursued. Lastly, Rashid et al. identified a ureteric-arterial-enteric fistula which was diagnosed postmortem via CT angiography after the patient's death from profuse pelvic haemorrhage [2].

## 5. Conclusions

In managing such complex patients, a multidisciplinary approach involving urology, oncology and general surgery is essential for optimal outcomes. The case was managed in the context of a multidisciplinary team (MDT), involving urology, general surgery, radiology and oncology, who jointly assessed the patient's surgical risk, radiological findings and functional status before opting for conservative management. In patients with previous pelvic surgery, radiotherapy and chronic stenting, delayed fistula formation may occur even in the absence of new oncological disease. This case highlights that conservative management can be successful, especially when the fistula resolves spontaneously and renal function is preserved. However, long-term follow-up, ongoing surveillance and shared decision-making with the MDT are essential. It is important to emphasise that individualised treatment, tailored to the patient's preferences and clinical condition, is crucial. Reporting such unique cases contributes to expanding our shared knowledge, improving surgical care, and we look forward to further research in this area.

## Figures and Tables

**Table 1:** Similar case reports.

**Citation**	**Age (years)/gender**	**Type of fistula**	**Findings**	**Management**	**Key learning point**
Mir et al. (2023) [7]	41 F	Uretero-jejunal fistula	The patient presented with pyelonephritis and hydronephrosis. She had a history of cervical cancer and had previously undergone a gastrojejunostomy for superior mesenteric artery syndrome. Subsequently, she developed a peptic ulcer at the jejunal site, which led to the formation of a uretero-jejunal fistula, ultimately resulting in acute pyelonephritis. The uretero-jejunal fistula was confirmed through nephrostogram and oesophagogastroduodenoscopy (OGD).	She was initially managed with a nephrostomy to divert urine away from the fistula and a percutaneous endoscopic gastrostomy (PEG) tube placed distal to the ulcer site to facilitate healing. A repeat OGD 6 months later demonstrated evidence of ulcer healing. Unfortunately, 1 year later, she presented with a perforated ulcer and passed away.	This case illustrates a rare uretero-jejunal fistula caused by a penetrating peptic ulcer in the setting of a gastrojejunostomy, with contributing factors including unneutralised acid exposure and prior chemoradiation. Initial conservative management with nephrostomy and distal PEG placement was successful, but the recurrence of ulcer perforation underscores the importance of long-term acid suppression and close follow-up.
Liu et al. (2022) [5]	70 M	Uretero-ileal fistula	The patient had a ureteric stent in his urostomy, which was accidentally dislodged. A new ureteric stent was inserted through the urostomy; however, an iatrogenic uretero-ileal fistula occurred due to intraoperative misplacement of the JJ stent using a Zebra guidewire. The stent perforated the ureter, with its proximal end entering the ileum. The double-J stent was subsequently passed in the feces 12 h postoperatively. The patient had a history of bladder cancer with a unilateral urostomy.	Patient passed before definitive management due to septic shock.	This fistula resulted from an intraoperative complication. Ureteric injury, although rare, is a recognised complication that can lead to fistula formation. Early recognition and prompt management are crucial in such cases. The patient's history of cancer likely contributed to tissue fragility. It is possible that the stent initially perforated the upper part of the ureter and was subsequently displaced into the bowel lumen by peristalsis.
Rashid et al. (2012) [2]	76 F	Ureteric-arterial-enteric fistula	Sudden large volume pelvic bleeding, either rectal or vaginal (known previous vesicovaginal fistula). Background of endometrial carcinoma surgically treated, with adjuvant radiotherapy.	Fistula was diagnosed postmortem, as patient passed due to massive blood loss.	The fistula potentially developed due to previous pelvic surgery and radiotherapy. This is a very unique case, where a ureteric-arterial-enteric fistula led to catastrophic lethal hemorrhage.
Chang et al. (1998) [3]	42 F	Ureteroenteric fistula	Upper ureteric-enteric fistula. Previous extracorporeal shock wave lithotripsy (ESWL) for renal stones 2 months prior.	Conservative management with JJ stent placement. Retrograde studies upon stent removal did not show extravasation of contrast.	This fistula may have arisen following ESWL. Successful conservative management with a ureteric stent which led to the fistula self-resolving. The stent was subsequently removed at a later stage.
Appleyard and Jones (1977) [4]	50 M	Uretero-ileal fistula	Distal uretero-ileal fistula after repair of a ureteric injury that happened during abdomino-perineal excision for rectal cancer. Patient had abnormal anatomy due to horseshoe kidney and ureters were located more medially.	Laparotomy and Boari flap reconstruction.	This case involves an iatrogenic fistula resulting from an intraoperative complication, which was subsequently managed successfully through surgical intervention in a second instance.
Blumgart and Thakur (1972) [6]	25 M	Uretero-ileal fistula	Uretero-ileal fistula with background of Crohn's disease.	Diagnostic laparotomy, where reimplantation of the healthy ureter and right hemicolectomy was carried out, with an end-to-end anastomosis between apparently the healthy ileum and colon.	This fistula arose as complication of underlying inflammatory bowel disease (Crohn's disease) and was successfully managed through surgical intervention.
Critchley (1967) [8]	87 F	Uretero-ileal fistula	A mid ureteric ileal fistula was seen with retrograde studies. Background of recurrent UTIs.	Diagnostic laparotomy and fistula closure with sutures. Later, nephro-ureterectomy was performed.	The fistula developed potentially due to chronic infections and ongoing local inflammation. Initial management involved surgical ligation of the fistula. However, the patient later represented with severe pyonephrosis, necessitating a nephroureterectomy.
Scott, (1965) [9]	52 F	Uretero-ileal fistula	A ureteric-distal ileal fistula was seen with retrograde studies. Investigations were done due to chronic flank and abdominal pain.	Nephro-ureterectomy, due to significant pyonephrosis, and small bowel resection were performed.	The aetiology of fistula formation in this case remains unclear, as the patient had no identifiable risk factors in their medical history. Surgical management involved resection of the affected kidney, ureter, and segment of small bowel.

## Data Availability

Data sharing not applicable to this article as no datasets were generated or analysed during the current study.
